# Public health training in public institutions’ speech-language-hearing programs in Northeastern Brazil: a curricular framework study

**DOI:** 10.1590/2317-1782/20232023042en

**Published:** 2023-12-18

**Authors:** Edicleide Martins da Silva, Ariela de Queiroz Correia Nóbrega, Janaína von Sӧhsten Trigueiro, Brunna Thais Luckwu de Lucena, Hosana Silmara Eleutério Silva, Eva Carolina Rezende Cruz, Luciana Figueiredo de Oliveira

**Affiliations:** 1 Programa Associado de Pós Graduação em Fonoaudiologia - PPGFON, Universidade Federal da Paraíba - UFPB - João Pessoa, PB, Brasil.; 2 Programa de Pós-Graduação em Saúde Coletiva - PPGSC, Universidade Federal da Paraíba - UFPB -João Pessoa, PB, Brasil.; 3 Departamento de Fonoaudiologia, Universidade Federal da Paraíba - UFPB - João Pessoa, PB, Brasil.; 4 Hospital Distrital de Solânea, Secretaria Estadual de Saúde da Paraíba - Solânea, PB, Brasil.

**Keywords:** Speech Therapy, Public Health, University Education, Academic Education, Public Health Education

## Abstract

**Purpose:**

To describe the characteristics of the initial public health training for speech-language-hearing therapists in Northeastern Brazil.

**Method:**

s: The research was based on the curricular framework of public higher education institutions in the Northeast. The analysis approached each institution’s general speech-language-hearing program data (state where it is located, total course load, and most recent curricular framework reformulation) and specific data on public health courses (their individual course load, the term when it is offered, syllabus content, whether it was theoretical, practical, both, or internship, and whether it was required or elective).

**Results:**

The data show that eight public higher education institutions in the Northeast offer speech-language-hearing programs, whose pedagogical frameworks date from 2009 to 2021. The total course load of those related to public health ranges from 7.5% to 20.5% of the total program among those analyzed. Most courses were elective, exclusively theoretical, and were offered in the first half of the program.

**Conclusion:**

The initial public health training for speech-language-hearing therapists in public higher education institutions in the Northeast still seems to be based on traditional practices. These create a distance between students, public health, and practices that meet the principles of Health Unic System (in Portuguese - Sistema Único de Saúde) and the population’s real needs, especially in primary healthcare

## INTRODUCTION

Since the National Curricular Guidelines^(1)^ were implemented in 2002, the speech-language-hearing (SLH) undergraduate programs have been concerned with reformulating their curricula to ensure that such therapists' initial training enables them to recognize and meet the population’s actual needs. Thus, they also emphasize the importance of increasing practical experience opportunities during undergraduate studies. These recommendations draw nearer the demands that health professionals face in primary healthcare, where they need to consider people’s real necessities according to the social, historical, and cultural context to which they belong^(2)^.

Thus, reviewing SLH curricula became necessary and urgent since SLH therapists were included among the health professionals in the teams at Family Health Support Centers - now named Extended Centers for Family Health and Basic Care. Regulation no. 2,436/2017^(3)^ determined that such professional teams should be defined by each municipal government, which, along with budget cuts for the Unified Health System (SUS, in Portuguese), weakened these centers and hindered the inclusion of SLH therapists (and other health professionals) - which, in turn, compromises users’ comprehensive healthcare.

Moreover, the existing gaps in SLH therapists’ initial training have also been pointed out among the factors that contribute to their little participation in primary healthcare and public policies^(4)^. Since the first days of this profession in Brazil, SLH therapists were accustomed to individual and private clinical practice, based on traditional training with an assistance mindset^(5-7)^.

Therefore, providing public health training to SLH therapists has an important role in regional development and may help include them in primary healthcare^(5)^. The scientific production on the topic has increased over the last few years. More recent studies were mainly based on the perception and experiences of undergraduate SLH students^(8,9)^ and professors^(4,10)^, with important contributions in the area, as they consider the opinion of subjects directly involved in and coauthors of the training process.

This research can broaden and strengthen reflections that are being made, considering an aspect that has been seldom approached - the analysis of undergraduate SLH curricular frameworks. Hence, the following question was raised: “What are the characteristics of the initial public health training for SLH therapists in Northeastern Brazil?”. To answer it, this study aimed to describe the characteristics of the initial public health training for SLH therapists in Northeastern Brazil based on the analysis of curricular frameworks in higher education institutions (HEIs).

## METHODS

This documentary research was conducted in April and May 2022, analyzing undergraduate SLH curricular frameworks at HEI in Northeastern Brazil. Since it did not involve humans, this research was exempted from obtaining informed consent.

In the first stage, the researchers consulted the list of HEIs on the website of the Federal SLH Council - 4^th^ Region (CREFONO 4), encompassing the states of Paraíba, Pernambuco, Alagoas, Sergipe, and Bahia, and 8^th^ Region (CREFONO 8), comprising Rio Grande do Norte, Ceará, Piauí, and Maranhão. It was verified that 21 undergraduate SLH programs were offered in 20 HEIs in the Northeast.

However, the SLH program was not found on two HEIs’ websites, and the platform of another HEI could not be accessed. Thus, the study addressed 18 Northeastern undergraduate SLH programs, distributed in 17 HEIs, as one of them offers the program on two campuses, with different curricular frameworks, structure, and faculty.

The second step was to research each HEI’s website, collecting their curricular framework, syllabus, and/or pedagogical framework. If the information necessary for the research was unavailable, an e-mail was sent to the HEI’s program coordinator requesting the said documents.

Information was collected from all public HEIs, but only one private HEI had summarized syllabi on the website. Moreover, the e-mails sent to the other HEIs were not replied to. Therefore, the analysis encompassed undergraduate SLH curricular framework data of eight public HEIs, which made up the study sample.

Each HEI’s documents were analyzed to obtain general data on its SLH program (state where it is located, total course load, and most recent curricular reformulation) and specific data on public health courses (their individual course load, the term when it is offered, syllabus content, whether it was theoretical, practical, both, or internship, and whether it was required or elective). However, this study did not analyze the course contents because they were available to the public in only a few HEIs.

The courses included in the analysis were defined by consensus among four of the researchers, who are public health professors and practitioners and, therefore, closely related to this area. Collected data were systematized in charts and descriptively analyzed.

## RESULTS

The courses are distributed per state as shown in Chart 1.

**Chart 1:** Distribution of public higher education institutions in Northeastern Brazil, according to the website of the Federal Speech-language-Hearing Council

State	N	%
Alagoas	1	12.5
Bahia	2	25.0
Paraíba	1	12.5
Pernambuco	1	12.5
Sergipe	2	25.0
Rio Grande do Norte	1	12.5
Ceará	0	0
Piauí	0	0
Maranhão	0	0
TOTAL	8	100%

**Caption:** N = Number.

The pedagogical framework of the SLH programs 1 and 6 was reformulated in 2009, which makes them the oldest SLH ones among the Northeastern public HEIs. Program 4 dates from 2015; program 5, from 2016; and program 7, from 2018. Programs 2 and 3 reformulated theirs in 2019, while program 8 has the most recent pedagogical framework among those analyzed, dating from 2021.

The mean total course load in undergraduate SLH programs in Northeastern public HEIs is 3,792 hours. The shortest program requires 3,015 hours, and the longest one totals 4,600 hours of activities to grant a bachelor’s degree in SLH Sciences.

As for public health, specifically, the data analysis approached the course load of both required and elective courses in this area. The percentage of hours in public health courses is shown in Chart 2.

**Chart 2:** Total and specific course loads in higher education institutions

**Code**	**Total course load**	**Public health course load**			
		**Elective**	**Optional**	**Total**	**%**
SLHP1	3.600 h	210 h	60 h	270 h	7.5
SLHP2	4.600 h	500 h	0 h	500 h	10.8
SLHP3	3.315 h	330 h	270 h	600 h	18.9
SLHP4	3.360 h	450 h	240 h	690 h	20.5
SLHP5	4.250 h	420 h	0 h	420 h	9.8
SLHP6	4.588 h	425 h	204 h	629 h	13.7
SLHP7	3.015 h	270 h	330 h	600 h	19.9
SLHP8	3.975 h	225 h	240 h	465 h	11.6

**Caption:** SLHP = speech-language-hearing program; h = hour.

In only one program, less than half of the public health courses were required. The analysis also approached the moment during the SLH program when required public health courses are offered, revealing that they are more prevalent in the first half of the program in most HEIs, as seen in Table 1.

**Table 1:** Total course load of elective courses and the moment when they are offered in each higher education institution

**Code**	**TOTAL COURSE LOAD OF ELECTIVE COURSES**		**COURSE LOAD 1/2**		**COURSE LOAD 2/2**	
	N	%	N	%	N	%
**SLHP1**	210 h	77.77%	120 h	57.14%	90 h	42.85%
**SLHP2**	500 h	100%	320 h	64%	180 h	36%
**SLHP3**	330 h	55%	180 h	54.54%	150 h	45.46%
**SLHP4**	450 h	65.2%	330 h	73.33%	120 h	26.67%
**SLHP5**	420 h	100%	345 h	82.14%	75 h	17.86%
**SLHP6**	425 h	65.56%	170 h	40%	255 h	60%
**SLHP7**	270 h	45%	150 h	55.55%	120 h	44.45%
**SLHP8**	225 h	48.38%	60 h	26.66%	165 h	73.33%

**Caption:** SLHP = speech-language-hearing program; h = hour.

**Figure 1:**
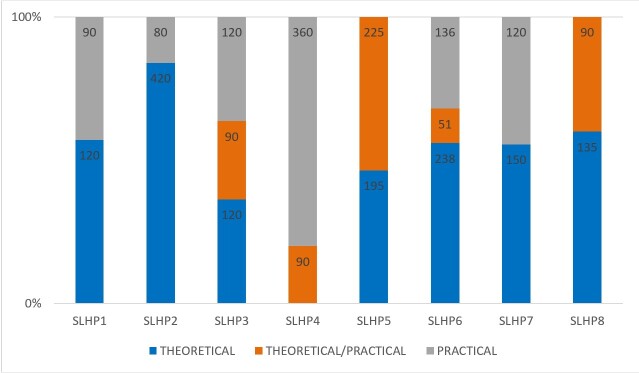
Characterization of required public health courses

As for the type of public health courses (whether they were exclusively theoretical, exclusively practical/internship, theoretical-practical, or internship), theoretical ones were predominant in five out of the eight programs analyzed, as shown in Figure 1.

## DISCUSSION

The data found in this study reveal that most undergraduate SLH programs in the Northeast underwent curricular reformulations in the last 7 years. Only two of them did so before that, as their pedagogical frameworks date from 2009.

Many programs had about 10 to 20% of their course load aimed at public health, divided into elective and optional ones. The SLH program 1 had the smallest course load focused on public health among all those analyzed. Moreover, this program’s curriculum is one of the oldest, which demonstrates that it must be urgently reformulated.

This information calls attention to the fact that most of the SLH therapists’ training ー about 80%, in the best perspective ー is still strongly based on SLH specialties focused on clinical/individual practices. Such a reality is recurrent^(4,10)^ in health programs overall, which (over)emphasize biomedical courses to the detriment of content related and committed to social and human training. This can be seen as a consequence of the capitalist ideology strengthened by the propagation of neoliberalism in Brazil^(11)^.

Hence, it is necessary to reflect on the aftermath of successive attacks on SUS and its budget cuts since 2017, particularly regarding primary healthcare. This scenario has directly affected Family Health Support Centers, which have been mischaracterized in many municipalities and are not working to their full potential. This has intensified the inversion/regression of the healthcare mindset, giving greater importance to individualized, uniprofessional, biomedical healthcare.

The inclusion of SLH therapists in SUS at Family Health Support Centers intensified and made urgent the need to consider and reformulate undergraduate SLH curricula. Nonetheless, it is likewise important to reflect that fragilizing this service - and, therefore, SLH therapists’ work in primary healthcare - may have consequences for educational practices. We may be witnessing a subtle and dangerous movement in the opposite direction of what has been proposed by the National Curricular Guidelines to SLH programs to train these therapists.

On the other hand, the recent GM/MS Regulation no. 635^(12)^, of May 22, 2023, aims to provide federal funds again to ensure a new version of multiprofessional primary healthcare teams, now named eMulti. These teams can mean a new opportunity to strengthen and qualify attention in primary healthcare, counting on more health professional categories among them, including SLH therapists. This intensifies the need for critical and differentiated training that once again opposes the medical-centered bias, which, for various reasons pointed out in this paper, insists on permeating SLH practices in primary healthcare.

Collected data also showed that in six out of these eight programs, more than half of the public health courses are offered in the first half of the program. Moreover, exclusively theoretical courses predominate in most HEIs. Providing curricular components in this format is not adequate for the SLH therapists’ training - it goes against what has been indicated in the National Curricular Guidelines^(1)^ and hinders committed and thoughtful practices, as expected in primary healthcare. Such a curricular organization may reflect the traditionalism still lingering in SLH educational practices, revealing that the Flexnerian model approached in 20th-century medical education still greatly influences health training.

These factors can be considered limitations to significant experiences in the process of training SLH students^(8,9)^. The strong presence of a biomedical bias in health professional training - particularly SLH therapists in this case - embodies the alienating process of technification and overspecialization on which higher education is traditionally based^(8-10)^.

SUS must not be reduced to its theoretical and legal aspects in health professional education; rather, it must be significantly approached and experienced in undergraduate studies. Meanwhile, current discussions aim to review and bring new meaning to SLH practices and training, change undergraduate curricular guidelines, and reformulate the students’ competencies and skills so they can view and experience health more comprehensively and effectively since their initial training.

Advancements have been made in required courses, as supposedly experienced thanks to the SLH curriculum reformulations analyzed in this study. However, students still have difficulties relating public health concepts to their practices, with a fragile integration between teaching, service, and the community - which is essential to significant learning^(9)^.

As a result of training in this format, professionals acquire a good theoretical basis but have difficulties dealing with the variety of real situations they face in everyday practice. Hence, the SLH therapists’ initial training must ensure opportunities to draw scientific knowledge nearer the activity they will carry out, creating a closer link between SLH knowledge and practices.

Since the National Curricular Guidelines^(1)^ were implemented in 2002, undergraduate SLH programs have been concerned with reformulating their curricula to ensure that their training enables them to recognize and meet people’s real needs. Hence, it is necessary to reflect on the various possibilities in which the SLH Sciences can and must be constructed/deconstructed.

Since the training guides the practice, it cannot be forgotten that it must be based on the population’s actual necessities - which makes the opposite also true, in that the practice guides the training. The idea is to see and place theory and practice side by side to dismiss the concept that crystalized scientific knowledge governs professional practices. Health professional training - focusing SLH therapists in this case - must approach healthcare according to the users’ particularities and demands, instead of scholarly constructed theoretical knowledge. Students/professionals must conform to practices/services - with no room for vice-versa in this sentence.

Public health courses, along with other ones that study human and social sciences, are usually seen as a possibility to change health professional practices. However, they are seemingly carried out quite distantly from the reality experienced in everyday work^(13)^. To meet what has been proposed in the National Curricular Guidelines^(1)^, the teaching-learning process must be redirected so that the pedagogical strategies lead to the students’ active participation in training.

The process of memorizing information and vertically transmitting fragmented knowledge must be replaced with practices that combine knowledge with an interprofessional approach^(14)^. Professionals cannot be expected to cooperate if their education is predominantly (or exclusively) uniprofessional.

Thus, Interprofessional Education in SLH therapists’ initial training is a powerful opportunity to add quality to their education. Discussions on this topic in the context of the SLH Sciences are still incipient, although they already point out the importance of interprofessional practices and highlight the invaluable contribution that the SLH Sciences can make to interprofessional communication^(15)^.

Studies have pointed out the urgency of using Interprofessional Education concepts in undergraduate health training^(4,16,17)^ to help future professionals ground their practices on such concepts and meet the population’s actual needs.

Supervised internship during initial SLH training is an important tool for future professionals^(2,18)^ to develop cooperative and interprofessional practices. Integration with other courses in the curricular framework tends to provide students with a humanized and generalist perspective capable of identifying cultural and social determinants of health-disease processes. Hence, it helps focus the SLH therapists’ practice on health promotion in all settings where they work.

Therefore, besides theoretical classes, SLH training must have a greater course load and possibilities of practical experiences in public health. SLH students cannot be expected to know and understand the needs and specificities in this field of knowledge if they are only superficially and technically presented to them.

Active methodologies are also considered an (urgent) need in SLH training. The concept of active methodology is to learn by doing - i.e., the threefold action-reflection-action transforms theoretical-practical aspects of knowledge acquisition and critical and reflexive construction of know-how^(19)^. Furthering this discussion and considering such data, it is important to highlight that the curricular framework of one of the programs analyzed in this study already approaches training based on active methodologies. This certainly enriches the teaching-learning process experienced by SLH students in that HEI.

Another point to emphasize is the barriers faced by professors, whose education was mostly based on traditional teaching practices and methodologies, in models that gave excessive importance to theoretical content. Therefore, in the training format still practiced in SLH Sciences, professors reproduce the models they received in their education, which are perpetuated in professional practice^(10)^. This demonstrates the need to (re)direct health professional training to meet the needs at SUS^(20-22)^.

Moreover, health and education public policies must be (re)formulated to transform health practices, which have remained crystallized, neglecting continuous changes in society. Thus, no more time can be spent on distant or shallow understanding and application of the concepts of health promotion and comprehensiveness, as they have been experienced in training and reproduced in SLH practices.

Resolution 07/2028 provides that at least 10% of undergraduate course loads be made up of public outreach programs. Accordingly, the current movement to include such programs in undergraduate health curricula may ensure significant experiences in SLH therapists’ initial training. The activities of public outreach programs at universities form an interdisciplinary, political, educational, cultural, scientific, and technological process capable of promoting a transforming interaction between HEIs and society, always coordinated with teaching and research^(23)^. Hence, such a movement can be seen as a support to provide SLH graduates/professionals with a generalist, humanist, critical, and reflexive profile.

This research could not analyze the syllabus of all public health courses, which is one of its limitations. Moreover, it cannot be stated that the reality presented here represents the SLH therapists’ initial public health training in the Northeast, as most HEIs that offer SLH programs in this region are private - whose data could not be analyzed because the data available on their websites were insufficient. This suggests the need for complementary research in the region.

Furthermore, as the training process must be always rethought, discussed, and adjusted, research such as this one must be conducted periodically to accompany changes made in curricular frameworks and their results.

## CONCLUSION

The collected data showed that SLH therapists’ initial public health training in Northeastern public HEIs is predominantly based on exclusively theoretical courses, mostly offered in the first half of the program. This configuration tends to distance students from the context of public health, possibly influencing their inclusion and practices in SUS according to its guiding principles, particularly regarding SLH practice in primary healthcare.
