# Supplementary material for: Public health training in public institutions’ speech-language-hearing programs in Northeastern Brazil: a curricular framework study
Source: Codas. 2023 Dec 18;36(2):e20230042. doi: 10.1590/2317-1782/20232023042en (PMC10750893; doi:10.1590/2317-1782/20232023042en)
Supplement: Translation

# Translation: A formação em saúde coletiva nos cursos de fonoaudiologia de instituições públicas do Nordeste: um estudo a partir das matrizes curriculares

**DOI:** 10.1590/2317-1782/20232023042pt

**Authors:** Edicleide Martins da Silva, Ariela de Queiroz Correia Nóbrega, Janaína von Sӧhsten Trigueiro, Brunna Thais Luckwu de Lucena, Hosana Silmara Eleutério Silva, Eva Carolina Rezende Cruz, Luciana Figueiredo de Oliveira

**Affiliations:** 1 Programa Associado de Pós Graduação em Fonoaudiologia - PPGFON, Universidade Federal da Paraíba - UFPB - João Pessoa, PB, Brasil.; 2 Programa de Pós-Graduação em Saúde Coletiva - PPGSC, Universidade Federal da Paraíba - UFPB -João Pessoa, PB, Brasil.; 3 Departamento de Fonoaudiologia, Universidade Federal da Paraíba - UFPB - João Pessoa, PB, Brasil.; 4 Hospital Distrital de Solânea, Secretaria Estadual de Saúde da Paraíba - Solânea, PB, Brasil.

**Keywords:** Fonoaudiologia, Saúde Pública, Ensino Superior, Formação Acadêmica, Educação em Saúde Pública

## Abstract

**Objetivo:**

Descrever as características da formação inicial em saúde coletiva de fonoaudiólogos do Nordeste do Brasil.

**Método:**

A pesquisa foi realizada com base nas matrizes curriculares de Instituições de Ensino Superior (IES) públicas do Nordeste a partir das quais foram analisados dados gerais dos cursos de Fonoaudiologia de cada Instituição ー estado em que está localizado, carga horária total e última reformulação de matriz curricular; e dados que diziam respeito especificamente às disciplinas relacionadas à Saúde Coletiva ー carga horária de cada disciplina, semestre de oferta, conteúdo da ementa, caráter teórico, prático, teórico-prático ou estágio, se obrigatória ou optativa.

**Resultados:**

Os dados revelam que existem oito IES públicas do nordeste que oferecem o curso de Fonoaudiologia, sendo que os Projetos Pedagógicos de Cursos datam de 2009 a 2021. A Carga Horária (CH) total das disciplinas vinculadas à área de Saúde Coletiva varia de 7,5% a 20,5% da CH total dos cursos analisados. A maioria destas disciplinas é eletiva, tem caráter teórico e é ofertada na primeira metade da formação.

**Conclusão:**

A formação inicial em saúde coletiva de fonoaudiólogos de IES públicas do nordeste parece ainda estar embasada em práticas tradicionais, que acabam distanciando os discentes do campo da saúde coletiva e de práticas que atendam os princípios do Sistema Único de Saúde, e as reais necessidades da população, especialmente na Atenção Primária à Saúde.

## INTRODUÇÃO

A partir da implantação das Diretrizes Curriculares Nacionais (DCN)^(1)^, em 2002, os cursos de graduação em Fonoaudiologia têm se preocupado em reformular seus currículos de forma a garantir que a formação inicial do fonoaudiólogo possibilite que o mesmo reconheça e atenda as reais necessidades da população, enfatizando também a importância do aumento das possibilidades de vivência práticas durante a graduação. Tais recomendações se aproximam das exigências para a atuação de profissionais de saúde na Atenção Primária à Saúde (APS), uma vez que neste contexto, é necessário que sejam consideradas as reais necessidades dos sujeitos a partir a realidade sócio-histórica e cultural nas quais eles estão inseridos^(2)^.

Por isso, desde que o fonoaudiólogo passou a integrar a equipe de profissionais de saúde que atuam nos então denominados Núcleos de Apoio à Saúde da Família (NASF), posteriormente denominados Núcleos Ampliado de Saúde da Família e Atenção Básica (NASF-AB), fez-se necessária e urgente a revisão dos currículos dos cursos de Fonoaudiologia. A deliberação da composição das equipes NASF-AB a cargo da gestão municipal imposta pela portaria 2.436/2017^(3)^, e o desfalque do financiamento do Sistema Único de Saúde (SUS), apresentaram como consequências o enfraquecimento desses Núcleos, dificultando a inserção do Fonoaudiólogos (e outros profissionais da saúde) e comprometendo integralidade do cuidado ao usuário.

Além disto, as lacunas existentes na formação inicial do fonoaudiólogo também têm sido consideradas um dos fatores que contribuem para a baixa inserção de fonoaudiólogos na APS e nas políticas públicas^(4)^. Isto porque desde o início da profissão no Brasil, este profissional estava habituado ao trabalho clínico individual e privado, a partir de uma formação tradicionalmente embasada por uma lógica assistencial^(5-7)^.

Entende-se, portanto, que a formação em saúde coletiva do fonoaudiólogo, além de ter importante papel no desenvolvimento regional, pode contribuir na inserção desse profissional na APS^(5)^. Nos últimos anos houve uma crescente produção científica relacionada ao tema. As pesquisas mais recentes foram realizadas sobretudo, a partir da percepção e experiências de discentes^(8,9)^ e docentes^(4,10)^ dos cursos de graduação em Fonoaudiologia, realizando importantes contribuições para a área, já que consideram a opinião de sujeitos diretamente implicados com e no processo de formação, podendo ser considerados coautores do mesmo.

A presente pesquisa pode ampliar e fortalecer as reflexões que já vem sendo realizadas, considerando um aspecto ainda pouco abordado: a análise das matrizes curriculares dos cursos de graduação em Fonoaudiologia. Portanto, questiona-se: quais as características da formação inicial em saúde coletiva de fonoaudiólogos na região nordeste do Brasil? Para respondê-la, este estudo tem o objetivo de descrever as características da formação inicial em saúde coletiva de fonoaudiólogos do nordeste do Brasil a partir da análise das matrizes curriculares das Instituições de Ensino Superior (IES).

## MÉTODO

Trata-se de uma pesquisa documental realizada nos meses de abril e maio de 2022, a partir da análise das matrizes curriculares dos cursos de Fonoaudiologia de IES localizadas no nordeste do Brasil. Por não envolver seres humanos a presente pesquisa dispensa a assinatura de Termo de Consentimento Livre e Esclarecido.

Na primeira etapa foi consultada a lista de IES existente no site do Conselho Federal de Fonoaudiologia - 4ª Região (CREFONO 4), que compreende os estados da Paraíba, Pernambuco, Alagoas, Sergipe e Bahia, e 8ª Região (CREFONO 8), composta pelos estados do Rio Grande do Norte, Ceará, Piauí e Maranhão, por meio da qual constatou-se a existência de 21 cursos de graduação em Fonoaudiologia em 20 IES na região nordeste.

Contudo, no site de duas IES, não foi encontrado o curso de Fonoaudiologia. Ademais, não se conseguiu acesso à plataforma de outra Instituição, restando portanto, 18 cursos de graduação em Fonoaudiologia situados no nordeste, distribuídos em 17 IES, já que uma das Instituições possui cursos em matrizes curriculares, estrutura e corpo docente diferentes em dois campos.

O segundo passo foi pesquisar o site de cada Instituição, captando-se das mesmas as matrizes curriculares, ementário e/ou Projeto Pedagógico do Curso (PPC). Salienta-se que para as Instituições que não apresentavam informações necessárias para a realização da pesquisa, foi enviado e-mail solicitando os documentos referidos aos coordenadores de curso.

Foi possível coletar informações de todas as IES públicas mas, no que tange às privadas, apenas uma apresentava ementas resumidas no site. Além disso, as pesquisadoras não obtiveram resposta aos e-mails enviados às demais Instituições. Por esse motivo, optou-se por analisar os dados das matrizes curriculares de cursos de graduação em Fonoaudiologia de oito IES públicas, sendo esse o número que representa a amostra do estudo.

A partir dos documentos procurou-se captar dados gerais dos cursos de Fonoaudiologia de cada Instituição ー estado em que está localizado, carga horária total e última reformulação de matriz curricular; e dados que diziam respeito especificamente às disciplinas relacionadas à Saúde Coletiva ー carga horária de cada disciplina, semestre de oferta, conteúdo da ementa, caráter teórico-prático, teórico-prático ou estágio, se obrigatória ou optativa. É importante salientar que para este estudo, não foram analisados os conteúdos programáticos das disciplinas em questão, pelo fato dos mesmos só estarem disponíveis para consulta pública em algumas IES.

Ressalta-se que as disciplinas analisadas foram as que apresentaram consenso entre quatro das pesquisadoras, docentes e profissionais atuantes na área de Saúde Coletiva e que, portanto, apresentam uma relação de pertencimento à área. Os dados coletados foram sistematizados em quadros e analisados descritivamente.

## RESULTADOS

No que diz respeito ao estado, os cursos estão distribuídos conforme o Quadro 1:

**Quadro 1:** Distribuição de IES públicas no nordeste de acordo com o site do CFFa

ESTADO	N	%
Alagoas	1	12,5
Bahia	2	25,0
Paraíba	1	12,5
Pernambuco	1	12,5
Sergipe	2	25,0
Rio Grande do Norte	1	12,5
Ceará	0	0
Piauí	0	0
Maranhão	0	0
TOTAL	8	100%

**Legenda:** N = número.

A respeito da reformulação dos Projetos Pedagógicos dos Cursos, é importante informar que o PPC dos cursos CF1 e CF6 datam de 2009, e são os mais antigos dentre os cursos de Fonoaudiologia das IES públicas do Nordeste. No CF4 o documento tem data de 2015, e no CF5 de 2016. Já o CF7 apresentou mudanças no PPC no ano de 2018. O CF2 e CF3 o fizeram no ano seguinte, em 2019 e o CF8 apresenta o PPC mais recente entre os cursos analisados, datando o ano de 2021.

No que se refere à carga horária (CH) total dos cursos de Fonoaudiologia das IES públicas do Nordeste, foi possível observar que a CH média dos cursos de Fonoaudiologia de IES públicas no Nordeste é de 3.792h, sendo que o curso com menor CH tem uma formação com 3015h e o que apresenta maior CH para a formação do bacharel em Fonoaudiologia totaliza 4.600h de atividades.

A respeito da CH das disciplinas voltadas à Saúde Coletiva, a análise dos dados teve como base as disciplinas obrigatórias e optativas relacionadas à essa área. A porcentagem da CH de disciplinas voltadas à Saúde Coletiva pode ser verificada no Quadro 2.

**Quadro 2:** Carga Horária total e específica nas IES

**Código**	**CH TOTAL**	**CH SC**			
		**Eletivas**	**Optativas**	**Total**	**%**
CF1	3.600h	210h	60h	270h	7,5
CF2	4.600h	500h	0h	500h	10,8
CF3	3.315h	330h	270h	600h	18,9
CF4	3.360h	450h	240h	690h	20,5
CF5	4.250h	420h	0h	420h	9,8
CF6	4.588h	425h	204h	629h	13,7
CF7	3.015h	270h	330h	600h	19,9
CF8	3.975h	225h	240h	465h	11,6

**Legenda:** CF = Curso de Fonoaudiologia; CH TOTAL = Carga Horária total; CH SC = Carga Horária Saúde Coletiva; h = Hora;

Percebeu-se que apenas em um curso as disciplinas obrigatórias representam menos da metade das disciplinas relativas à Saúde Coletiva. Além disso, também foi analisado o momento de oferta das disciplinas obrigatórias voltadas à Saúde Coletiva durante a graduação em fonoaudiologia, sendo evidente que na maioria das Instituições as mesmas são mais prevalentes na primeira metade dos cursos, como pode ser observado na Tabela 1.

**Tabela 1:** Carga Horária total das disciplinas eletivas e momento de oferta das mesmas em cada IES

**Código**	**CH TOTAL DISCIPLINAS ELETIVAS**		**CH 1/2**		**CH 2/2**	
	N	%	N	%	N	%
**CF1**	210h	77,77%	120h	57,14%	90h	42,85%
**CF2**	500h	100%	320h	64%	180h	36%
**CF3**	330h	55%	180h	54,54%	150h	45,46%
**CF4**	450h	65,2%	330h	73,33%	120h	26,67%
**CF5**	420h	100%	345h	82,14%	75h	17,86%
**CF6**	425h	65,56%	170h	40%	255h	60%
**CF7**	270h	45%	150h	55,55%	120h	44,45%
**CF8**	225h	48,38%	60h	26,66%	165h	73,33%

**Legenda:** CF = Curso de Fonoaudiologia; CH = Carga Horária; h = Hora.

De acordo com o caráter das disciplinas voltadas à Saúde Coletiva (exclusivamente teórica, exclusivamente prática/estágios, teórico-práticas, estágios), constatou-se a predominância de disciplinas teóricas em cinco dos oito cursos analisados, como exibido na Figura 1.

**Figura 1:** Caracterização das disciplinas obrigatórias de saúde coletiva

## DISCUSSÃO

Os dados encontrados nesse estudo revelam que a maioria dos cursos de graduação em Fonoaudiologia no Nordeste passou por reformulações curriculares nos últimos sete anos. Sendo que apenas dois deles o fizeram anteriormente, tendo seus PPCs datados de 2009.

No que diz respeito à CH voltada às disciplinas relacionadas à Saúde Coletiva percebe-se que grande parte dos cursos possui cerca de 10 a 20%. Ressalta-se que essa CH é dividida entre disciplinas eletivas e optativas. O CF1 apresenta a menor carga horária destinada à Saúde Coletiva entre os cursos analisados. No entanto, enfatiza-se que este curso apresenta também um dos currículos mais antigos entre os analisados, evidenciando a urgência na reformulação do mesmo.

Tais informações chamam atenção à medida que é possível inferir que grande parte da formação do fonoaudiólogo ー cerca de 80%, na melhor perspectiva ー ainda tem se baseado fortemente nas especialidades fonoaudiológicas que tem foco no fazer clínico/individual. Uma realidade frequente^(4,10)^ encontrada nos cursos de saúde, de forma geral, nos quais se observa a [super] valorização de disciplinas biomédicas em detrimento de conteúdos relacionados e comprometidos e com uma formação social e humana, o que pode ser entendido com uma consequência da ideologia capitalista fortalecida com a difusão do neoliberalismo no Brasil^(11)^.

Nesse sentido, torna-se imperativo refletir a respeito das consequências dos sucessivos ataques e desfalques quanto ao financiamento que o SUS, e mais especificamente, a APS, sofreram a partir de 2017. Esse cenário tem repercutido diretamente no NASF, que em muitos municípios está descaracterizado e não tem tido possibilidade de funcionar com toda a sua potência. Em decorrência desse processo, tem-se vivenciado a intensificação da inversão/regressão na lógica de cuidado, que valoriza atendimentos individualizados, uniprofissionais e com caráter biomédico.

Se é possível considerar que a inserção do fonoaudiólogo no SUS a partir do NASF intensificou e fez urgente a necessidade de reflexões e reformulações nos currículos dos Cursos de graduação em Fonoaudiologia, é preciso refletir também como o enfraquecimento desse serviço, com consequente fragilização da atuação do fonoaudiólogo na APS, pode acabar repercutindo nas práticas formativas. Se entende portanto, que podemos estar acompanhando um discreto e perigoso movimento que vai na contramão do que as DCNs dos cursos de Fonoaudiologia tem proposto para a formação dos fonoaudiólogos.

Cabe destacar, no entanto, a recente portaria GM/MS Nº 635^(12)^, de 22 de maio de 2023, que foi lançada com a proposta de garantir novamente o incentivo financeiro federal para uma nova versão de equipes Multiprofissionais na APS, a partir das agora denominadas eMulti. Tais equipes podem significar uma nova oportunidade de fortalecer e qualificar o cuidado prestado na APS, com a presença de mais categorias profissionais de saúde atuando nesse contexto, entre eles, o fonoaudiólogo. O fato intensifica a necessidade de uma formação crítica e diferenciada, que mais uma vez, vá de encontro ao viés medicalocêntrico que, por diversos motivos abordados nesse texto, insiste em permear as práticas fonoaudiológicas na APS.

A partir dos dados coletados, constatou-se também que em seis dos oito cursos analisados, mais da metade das disciplinas do campo da Saúde Coletiva são ofertadas na primeira metade da formação. Não obstante, atenta-se para o fato de que na maioria das instituições há uma predominância de disciplinas exclusivamente teóricas. A oferta de componentes curriculares nesses moldes não são adequados para a formação do fonoaudiólogo, indo de encontro ao que tem preconizado as DCN^(1)^ e ainda, dificultando uma atuação mais implicada e reflexiva, como se espera na APS. Entende-se que tal organização curricular pode ser reflexo da representação do tradicionalismo ainda presente nas práticas formativas dos fonoaudiólogos, e evidenciam que o modelo flexneriano, proposto para o ensino médico no século XX, ainda exerce grande influência na formação em saúde.

Tais fatores podem ser considerados limitações para uma experiência significativa no processo formativo dos estudantes de Fonoaudiologia^(8,9)^. Nesse sentido, considera-se que a forte presença do viés biomédico na formação do profissional de saúde e, aqui especialmente do fonoaudiólogo, dá corpo ao processo alienante de tecnificação e superespecialização no qual o ensino superior tradicionalmente tem se baseado^(8-10)^.

Entende-se a necessidade de que durante a formação profissional de saúde, o SUS não seja reduzido aos seus aspectos teóricos e legais, mas que seja abordado e vivenciado de forma significativa durante sua graduação. Nesse ínterim, atualmente estão sendo realizadas discussões que objetivam rever e ressignificar as práticas e formação em Fonoaudiologia, partindo de mudanças nas diretrizes curriculares da graduação, passando pela reformulação das competências e habilidades dos estudantes a fim de que a saúde seja entendida e vivenciada, desde a formação inicial, de maneira mais abrangente e eficaz.

É preciso ainda atentar para o fato de que a despeito do avanço na oferta de componentes curriculares obrigatórios, como acredita-se estar vivenciando a partir da reformulação dos currículos dos cursos de Fonoaudiologia aqui analisados, os discentes continuam apresentando dificuldades em relacionar os conceitos da Saúde Coletiva com a própria atuação e fragilidades na integração ensino-serviço-comunidade, entendido como fundamental para uma aprendizagem significativa^(9)^.

Em decorrência de uma formação nesses moldes, tem-se profissionais com boa base teórica, mas com bastante dificuldade em lidar com situações reais e diversificadas em seu fazer cotidiano. Perante essa realidade, é imprescindível que, durante a formação inicial do fonoaudiólogo, sejam geradas oportunidades que possibilitem a aproximação entre o conhecimento científico e a atividade que o profissional desenvolverá, suscitando maior intimidade entre os saberes e fazeres fonoaudiológicos.

Desde a implantação das DCN^(1)^, em 2002, os cursos de graduação em Fonoaudiologia têm se preocupado em reformular seus currículos de forma a garantir que a formação deste profissional possibilite que o mesmo reconheça e atenda às reais necessidades da população. É imperativo refletir sobre as diversas possibilidades em que a Fonoaudiologia pode e precisa ser (des)construída.

Ao considerar a formação como norteadora da prática, não se pode esquecer que essa deve estar embasada nas reais necessidades da população, e claro, que em uma via de mão dupla, a prática norteia a formação. Trata-se de olhar e colocar a teoria e prática lado a lado, na tentativa de pôr um fim na ideia de que o saber científico e cristalizado deve reger as práticas profissionais. Na formação dos profissionais de saúde, e aqui em especial, do fonoaudiólogo, deve-se considerar o cuidado a partir das particularidades e demandas dos usuários, e não a partir do saber teórico construído na academia. O estudante/profissional deve moldar-se à prática/serviço e o vice-versa não existe nessa frase.

Apesar das disciplinas de saúde coletiva, juntamente com as que abordam estudos a respeito das ciências humanas e sociais serem geralmente vistas como possibilidade de mudança nas práticas dos profissionais de saúde, a condução das mesmas parece estar distante da realidade vivenciada durante a atuação^(13)^. Para atender a propositura das DCNs^((1))^, é necessário que haja uma reorientação do processo de ensino-aprendizagem, de modo que as estratégias pedagógicas impliquem na participação ativa dos estudantes em sua formação.

É necessário substituir os processos de memorização de informações e transferência fragmentada de saberes na forma verticalizada para uma prática que reúna saberes por meio de uma postura interprofissional^(14)^. Afinal, não é possível esperar que os profissionais atuem de forma colaborativa utilizando práticas formativas predominantemente (ou até, exclusivamente) uniprofissionais.

Nesse sentido, a Educação Interprofissional (EIP) na formação inicial do fonoaudiólogo tem aparecido como uma potente possibilidade para agregar qualidade a esta formação. A esse respeito, as discussões na Fonoaudiologia ainda podem ser consideradas discretas, mas já revelam a importância das práticas interprofissionais e destacaram a valiosa contribuição que a Fonoaudiologia pode apresentar, considerando a comunicação interprofissional^(15)^.

A utilização dos conceitos da EIP está sendo apontada como urgente na formação dos cursos de graduação da área de saúde^(4,16,17)^, no intuito de que os futuros profissionais possam fundamentar suas práticas em tais conceitos com vistas a atender as reais necessidades da população.

Os estágios supervisionados realizados durante a formação inicial do fonoaudiólogo se constituem como uma importante ferramenta para futuros profissionais^(2,18)^ exercerem práticas colaborativas e interprofissionais. A integração com as demais disciplinas da matriz curricular tende a proporcionar ao corpo acadêmico um olhar humanizado e generalista, capaz de identificar os determinantes culturais e sociais dos processos de saúde-doença, favorecendo que a práxis do fonoaudiólogo também possua o enfoque na promoção da saúde em todos os âmbitos de atuação.

Assim, destaca-se a necessidade de, para além de disciplinas teóricas, aumentar a CH e as possibilidades de vivências práticas no campo da Saúde Coletiva durante a formação do fonoaudiólogo. Não há como esperar que o estudante de Fonoaudiologia conheça e entenda as necessidades e especificidades deste campo de saber, apresentando-o de maneira superficial e tecnicista.

A utilização de metodologias ativas também tem sido considerada uma necessidade (urgente) na formação em Fonoaudiologia. A conceitualidade da metodologia ativa é o aprender fazendo. É a partir da tríplice “ação-reflexão-ação” que ocorrerá uma transformação nos aspectos teóricos-práticos da aquisição do conhecimento e na construção crítica e reflexiva do saber^(19)^. Fortalecendo a discussão em comento, levando este dado em consideração, cabe destacar que a matriz curricular de um dos cursos analisados já traz a proposta de formação baseada em metodologias ativas, o que certamente, enriquece o processo de ensino-aprendizagem vivenciado pelos estudantes de Fonoaudiologia da referida instituição.

Convém enfatizar também os entraves encontrados pelos próprios docentes que, em sua maioria, tiveram seus processos formativos baseados em práticas e metodologias tradicionais de ensino, com a supervalorização do modelo conteudista. Dessa forma, o modelo de formação que ainda hoje é vivenciado na Fonoaudiologia, os docentes repassam os modelos aprendidos na sua formação, e tais modelos são reproduzidos na atuação profissional^(10)^. Fica evidente, portanto, a necessidade de (re) orientar a formação dos profissionais da saúde a fim de responder às necessidades do SUS^(20-22)^.

Defende-se ainda também a necessidade de (re)formulação de políticas públicas de saúde e de educação, que possibilitem a transformação das práticas de saúde, que, negligenciando as mudanças continuamente ocorridas nas sociedades, têm-se mantido cristalizadas. Assim, não há mais tempo para distanciamento ou mesmo entendimento e aplicação rasos dos conceitos de Promoção e Integralidade da Saúde, como tem-se vivenciado na formação e reproduzido nos cenários de prática fonoaudiológica.

O atual movimento de inserção curricular da extensão universitária nos cursos de graduação em saúde, conforme estabelece a Resolução 07/2028, que prevê que na composição da CH dos cursos de graduação sejam compostas por no mínimo 10% de atividade de extensão pode possibilitar experiências significativas na formação inicial do fonoaudiólogo. As atividades de extensão universitária constituem-se em processo interdisciplinar, político educacional, cultural, científico, tecnológico, que são capazes de promover a interação transformadora entre as IES e a sociedade, devendo sempre estar articuladas ao ensino e a pesquisa^(23)^. Nesta direção, entende-se que tal movimento pode ser visto como um suporte para que tenhamos um perfil do formando egresso/profissional o Fonoaudiólogo, com formação generalista, humanista, crítica e reflexiva.

Ressalta-se que na presente pesquisa não foi possível analisar as ementas de todas as disciplinas relacionadas com o campo da saúde coletiva, o que representa uma das limitações deste estudo. Não obstante, também não é possível informar que essa representa a realidade da formação inicial em Saúde Coletiva dos fonoaudiólogos graduados no nordeste, tendo em vista que a maioria das IES que oferecem o curso de fonoaudiologia nesta região são privadas, e que não foi possível analisar os dados das mesmas, por insuficiência de informações publicizadas pelas mesmas em seus respectivos sites, o que sugere a necessidade de uma pesquisa complementar na região.

Além disso, entendendo que o processo de formação deve estar sempre sendo alvo de reflexões, discussões e ajustes, faz-se necessário ainda que pesquisas como esta sejam realizadas periodicamente, a fim de acompanhar as mudanças realizadas nas matrizes curriculares e seus resultados.

## CONCLUSÃO

A partir dos dados obtidos verificou-se que a formação inicial em Saúde Coletiva dos fonoaudiólogos em IES públicas do nordeste está, predominantemente, baseada em disciplinas de caráter exclusivamente teórico, ofertadas, em sua maioria, na primeira metade do curso de graduação. Essa configuração tende a distanciar os discentes do âmbito da Saúde Coletiva e, consequentemente, pode influenciar em sua inserção e na realização de práticas que atendam aos princípios norteadores do SUS, sobretudo quando se considera a atuação fonoaudiológica na APS.
